# The ailments that stem from cheese and relevant precautions taken in the Ottoman Empire from the 19th century to the 20th century

**DOI:** 10.1002/fsn3.3849

**Published:** 2023-11-21

**Authors:** Ayşe Erkmen, Nevim Tüzün, Osman Erkmen

**Affiliations:** ^1^ Atatürk Principles and Revolution History Department Gaziantep University Gaziantep Turkey; ^2^ Department of History, Faculty of Human Sciences Ardahan University Ardahan Turkey; ^3^ Department of Nutrition and Dietetics, Faculty of Health Sciences İstanbul Arel University İstanbul Turkey

**Keywords:** cheese spoilage, disinfection, foodborne disease, hemlock, Ottoman archive

## Abstract

The increase in cheese production, sale, and consumption due to the settled lives of societies has led to an increase in cheese‐related diseases. It has become essential to better understand cheese‐borne diseases and to develop control measures. In this study, cheese‐related diseases and precautions taken in the Ottoman Empire from the 19th to the 20th centuries were investigated in Ottoman archival sources. Of these documents, cheese spoilage was detected in 12 and cheese‐related disease in 9. Cheeses that caused diseases or disorders in the relevant period were called spoiled cheese. One document states that a person died of a cheese‐borne illness. Cheese poisoning occurs mainly from unsalted, fresh cheeses. It has been determined that tin‐free copper pots were used in cheese production and sales and covered with herbs such as hemlock during maturation. In the relevant period, microbiological and chemical analyses of cheeses in terms of health were carried out in food control laboratories. Since the mercury chloride solution is used to disinfect animal udders, it has been stated that it contaminates the milk used in cheese production. Authorities have requested a boric acid solution (5% boric acid in hot water) instead of this solution for udder disinfection. In the Ottoman Empire, it was requested to take necessary sanitation and hygiene measures to prevent spoilage and cheese‐related diseases in the production areas or sales places of cheese. Clean and tinned containers should be used in cheese production, storage, and sale, and poisonous herbs should not be used during cheese ripening. It was also essential to analyze them in laboratories at internal and external customs. Knowing the precautions taken in the past to prevent the deterioration of food or the occurrence of diseases has led to modern food safety practices being applied today.

## Graphical abstract

Cheese spoilage and cheese‐related diseases have been detected in Ottoman documents. One document states that a person died of a cheese‐borne illness. Clean and tinned containers were used in cheese production, storage, and sale, and poisonous herbs should not be used during cheese ripening.

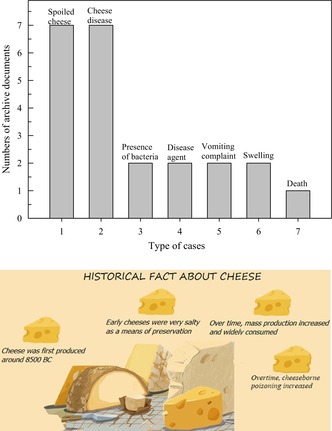

## 1 INTRODUCTION

In present‐day northwestern Türkiye, milk was obtained from animals, and dairy products, such as cheese, were manufactured 8500 years ago (Erkmen & Bozoglu, 2016a). Byzantine, Roman, Seljuk, and Ottoman people produced traditional dairy products, especially cheese, in the geography subject to the research, and this process continues to develop in the Republic of Turkey. As people got to know new settlements and met new neighbors, cheeses became more diversified, with traditional cheeses unique to the regions (Gerolymatou, 2016; Koçak, 2009; Mercer, 2021). The transition to settled life has enabled it to be consumed in more distant settlements. This has led to an increase in cheese spoilage and disease (Mercer, 2021). There is a growing recognition that the process from the past to the present needs to be known to better understand foodborne diseases and develop control measures. By knowing the cheese production technologies, cheese‐borne diseases, and precautions taken from the past to the present, technologies can be created and effective measures can be taken to produce healthier cheese today. Societies can form better ideas about today by understanding the way of life and nutrition of yesterday.

Milk has been an important part of nutrition for many creatures in nature since birth. Cheese has come to the forefront among dairy products because it can be preserved for a long time. So much so that cheese, which started to be produced in different shapes and flavors over time, has become one of the symbols of the cultural richness of nations and their transition to civilization (Saygılı et al., 2020). Figures show the production of cheese in the paintings and inscriptions dating back to 7000–10,000 BC found in temples in Mesopotamia (Durlu & Gün, 2007).

Food poisoning usually begins between 30 min and 72 h after consumption of food contaminated with pathogens, usually with sudden nausea and vomiting. Other symptoms of food poisoning include diarrhea, severe abdominal pain, abdominal cramps, and sometimes fever (Erkmen & Bozoglu, 2016b). The World Health Organization (WHO), in its report on food safety published in 2022, stated that approximately 1 in 10 people get sick from contaminated food every year and approximately 420,000 people die annually (WHO (Worl Health Organization), 2022). WHO also reported that unsafe food can cause food poisoning and diseases such as cancer, strain health systems, and hinder development by damaging national economies. Before the 20th Century, when awareness of microorganisms was not widespread, analysis methods for microorganisms were not used effectively, and foodborne poisoning was not emphasized. It is important to have information about the situation of food poisoning and what kind of measures were taken for food safety from the past to the present. Because of the change in cheese production methods in the process, the diversification of cheeses and the efforts to deliver cheese to more people due to the increase in production have led to the spread of cheese‐borne diseases. States took measures against these dangers. There has not been enough research on the records of food spoilage and foodborne diseases in the Ottoman Empire. This study was aimed at investigating the situation of cheese‐borne diseases in Ottoman archive (official) documents from the 1800s to the early 1900s since cheese was widely consumed in the Ottoman Empire and could adversely affect health when it was not produced and sold under appropriate conditions. From research on past food poisoning, we can obtain information such as the identification and analysis of facts about food poisoning, how food poisoning affects public health, how current practices on food safety are developed, and the precautions taken regarding these. We learn how some positive methods that were implemented in the past can be implemented, how today's modern food preservation techniques can be used better, and how public health has been protected from food poisoning from history to the present. The identification and compilation of early food poisonings and practices will also provide a unique opportunity to apply a range of scientific methods that will help further elucidate regional/social habits and patterns and resolve imbalances and limitations in the historical evidence. It is also aimed at determining the importance of the measures taken against the cheese diseases seen in the past in the development of food safety by revealing the official documents. In addition, the legal regulations made by the Republic of Turkey in the early period of the Republic of Turkey are also included.

## 2 METHOD

### 2.1 Place of archive documents

The official books, registers, and documents containing the correspondence and decisions taken by the elements constituting the central and provincial organization of the Ottoman Empire were kept in archives (Aktaş & Halaçoğlu, 1992). Today, the Republic of Turkey preserves these documents in the Prime Ministry Ottoman Archives (BOA).

### 2.2 Research in the archive

At the beginning of the archival study, research was conducted between October 2022 and March 2023. A comprehensive literature research on the subject was conducted. Afterwards, the BOA archives, which constitute the main source of the study, were searched. As a result of the search, it was determined that there are many documents titled Cheese in the archive. For this study, the archives were searched using the Turkish words “cheese,” “bad cheese,” “cheese spoilage,” “souring,” “dairy products,” “cheese poisoning,” and “poisoning”.

### 2.3 Study on the archive documents

After examining the documents identified as a result of the scanning in the archive, 39 documents suitable for the study were identified and transcribed from Ottoman Turkish to modern Turkish, and then another examination was made. As a result of the examination, it was determined that 31 of the 39 documents transcribed were suitable for the subject of the study. While 23 of these documents were related to cheese spoilage, cheese‐related diseases, and production and sales conditions, 8 of them were related to cheese prices and fines for selling spoiled cheese. The documents that are the subject of the study include the correspondence between Ottoman official institutions.

### 2.4 Identified archive documents

These documents are identified in the BAO: 68‐63/YB.021; 80‐36/YB.021; 90‐357/YB.021; 668‐54/HR.SFR.04; 2490‐50/DH.MKT; 2503‐22/DH.MKT; 704‐63/DH.MKT; 704‐63/DH.MKT; 242‐112/Y.MTV; 301‐67/ZB; 301‐51/ZB; 225‐10/HR.SFR.04; 15‐79/Y.PRK.MK; 294‐19/HR.TH; 813‐35/DH.MKT; 958‐32/DH.MKT; 2388‐179045/BEO; 913‐42/DH.MKT; 674‐99/HR.SFR.04; 827‐70/DH.MKT; 109‐58/YB.021; 9‐864/TFR.I.UM; 80‐3965/C.BLD; 31‐1533/C.BLD; 76‐3792/C.ZB; 310‐21/MVL; 67‐34/YB.021; 68‐170/YB.021; 72‐166/YB.021; and 4‐113/YB.021. Within the scope of the subject, literature research was conducted, supported by sources, and the article was started to be written. The article written in today's Turkish has been completed in English.

### 2.5 Statistical analysis

**FIGURE 1:**
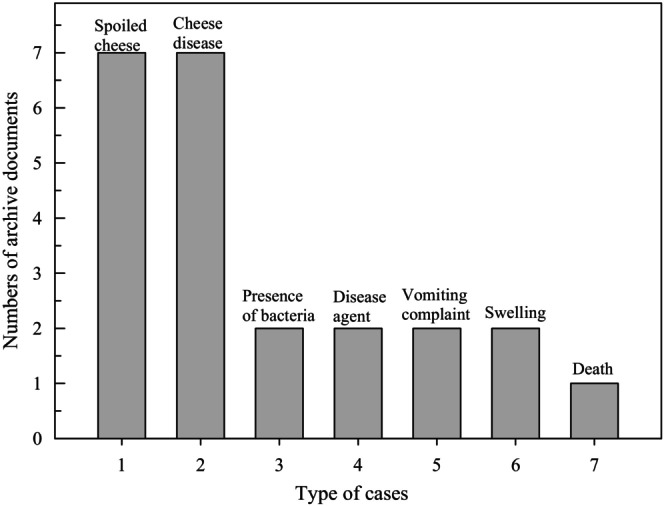
Number of archive documents about types of cases.

The number of archive documents indicating the idea in Figure 1 was analyzed using SPSS Statistics v.22 (IBM SPSS Corporation, Chicago, IL, USA). The means of the results were evaluated with significant differences (*p* < .05).

## 3 RESULTS

In the documents found in the BAO, the calendar abbreviations are as follows: R = Rumi, H = Hijri, and M = Miladi. In the documents, the measure of weight was used as okka = 400 dirhams or 1282 g, and the currency unit as one kurus = 40 coins.

### 3.1 Cheese diseases and spoilage in the Prime Ministry Ottoman Archives (BOA)

*June 8, 1872 Dated Archival Document*: This document states that the municipality of Skopje in Macedonia imposed 12 fines on sellers who sold unsanitary (spoiled) cheese (BOA, 68‐63).

*August 22, 1883 Dated Archival Document*: This document states that a grocery store in Skopje Municipality in Macedonia was fined for spilling cheese juice on the square (BOA, 78‐69).

*April 21, 1885 Dated Archival Document*: This document states that some cheeses sold in Skopje Municipality in Macedonia were defective, not suitable for consumption, and could cause disease when eaten, and that samples were taken and sent to the laboratory for analysis (BOA, 80‐36).

*June 29, 1891 Dated Archival Document*: This document states that the cheeses sold in a grocery store in Skopje Municipality in Macedonia were spoiled, that they could cause disease, and that municipal authorities should inspect these cheeses (BOA, 90‐357).

*October 13, 1892 Dated Archival Document*: In this document, it was stated that the cheeses to be exported were sent back to their place of origin on the grounds that they were spoiled (BOA, 668‐54).

*May 28, 1901 Dated Archival Document*: This document states that some people in İstanbul became ill after eating fresh meadow cheese, and they were taken under treatment. It was requested to investigate in detail the conditions under which the cheeses that caused the disease were produced and offered for sale (whether the containers in which the cheese was produced were tinned, whether healthy conditions were created in production, and whether pre‐sale inspections were carried out). The document also stated that cheeses should be produced in tinned copper containers (BOA, 2490‐50). In this document, it was reported that cheeses should be checked for compliance with health conditions at provincial customs.

*June 25, 1901 Dated Archival Document*: This document states that cheeses produced in some provinces of the country were brought to İstanbul, that people who ate these cheeses fell ill, and that these people were treated. It was reported that these cheeses were offered for sale at customs without being checked for health. The document states that unhealthy brined curd cheese, kashar cheese, Şarköy kashar cheese, and salty cheeses were brought to İstanbul (BOA, 2503‐22).

*October 9, 1901 Dated Archival Document*: This document states that fresh meadow cheese was brought to İstanbul from the province of Tokat without customs analysis and that controls and analysis should be carried out to determine whether these cheeses were spoiled or not (BOA, 2541‐118).

*May 9, 1903 Dated Archival Document*: This document states that people who ate fresh meadow cheese sold by a cheesemonger in the Beşiktaş district of İstanbul fell ill and applied to the İstanbul Naval Hospital, and were treated. It was reported that it was determined that these people were sickened by the cheese, and the sale of the cheese was stopped. It was stated that samples of the cheese were taken and sent for analysis, and bacteria were detected in the cheese. It was reported that the cheeses producing dairy should be checked (BOA, 704‐63).

*April 18, 1903 Dated Archival Document*: This document states that two people fell ill after eating fresh meadow cheese purchased from a grocery store in the Eyüp neighborhood of İstanbul and were treated at the Regimental Medical Hospital. It has been reported that necessary investigations were initiated against the sellers and cheese producers, and fines were given (BOA, 242‐112).

*April 22, 1903 Dated Archival Document*: This document states that those who ate the cheeses sold in İstanbul became ill and vomited, and that there were complaints that the cheeses were spoiled. It is stated that municipal officials took 200 grams of these cheeses and sent them to a laboratory for bacteriological and chemical (such as lead) analysis. It was also reported that 300 grams of these cheeses were fed to an experimental animal, and the results were analyzed. As a result of the analysis, the cheeses were deemed suitable for consumption (BOA, 301‐67).

*May 21, 1903 Dated Archival Document*: This document states that a cheese seller paid the required fee and asked for his cheeses to be analyzed for health and that the cheeses were reported to be fit for consumption as a result of the analysis (BOA, 301‐51).

*August 25, 1903 Dated Archival Document*: This document states that the cans (16 kg aluminum cans) brought from Bulgaria and containing brine cheese swelled at the points of sale. It was stated that measures should be taken at the Bulgarian customs regarding this issue (BOA, 225‐10).

*September 7, 1903 Dated Archival Document*: This document states that swelling was detected on the cans of cheese brought from Bulgaria; therefore, it was concluded that the cheeses were spoiled and measures should be taken at the Bulgarian customs (BOA, 15‐79).

*November 16, 1903 Dated Archival Document*: This document states that 19 sacks of cheese were imported from the Varna strait, and customs officials took samples of the cheese and sent them to the laboratory to check whether there were bacteria and chemicals harmful to health (BOA, 294‐19).

*January 31, 1904 Dated Archival Document*: This document states that the desired rennet produced from the stomachs of lambs and similar small cattle in Germany was analyzed (to determine whether it was tainted or not), and it was decided that there was no harm in importing and using it according to the results of the analysis (BOA, 813‐35).

*February 10, 1904 Dated Archival Document*: This document states that some people who ate fresh meadow cheese in İstanbul fell ill; the udders of the animals from which the milk used in cheese production was obtained were not cleaned properly; and after the cheese was produced, it was covered with hemlock (*Conium maculatum*, a highly poisonous herbaceous flowering plant in the carrot family). It was reported that they tried to protect them from drying by covering them with leaves. It was stated that cheeses produced in this way would carry a risk of disease, and such production should not be allowed (BOA, 958‐32).

*August 11, 1904 Dated Archival Document*: This document states that a person who ate cheese sold in a shop in Söke district of Aydın province fell ill and died (BOA, 2388‐179045).

*December 13, 1904 Dated Archival Document*: This document states that most of the Balkan cheeses brought from Beirut to Damascus were spoiled, and those that were not spoiled deteriorated within 15 days, that this deterioration started 2 months ago, and that the cheeses should be checked by chemists at the provincial customs (BOA, 913‐42).

*October 29, 1904 Dated Archival Document*: This document states that the Veterinary Medicine of Sofi analyzed the white cheeses in Sofia (Bulgaria), and that the cheeses were deemed fit for health and could be sold in İstanbul (BOA, 674‐99).

*May 7, 1905 Dated Archival Document*: This document states that three people aged 25 years and a person aged 35 years got sick after eating fresh meadow cheese brought from Izmit province and sold in İstanbul Bakırköy district. They vomited and were treated at the hospital. It has been reported that the udders of the animals were disinfected with a mercury chloride (HgCl_2_) solution, and the cheeses were covered with hemlock. As a result of the laboratory analysis of cheeses, mercury and hemlock poison were determined. Poisoning has been reported to be caused by hemlock and mercury. The document also states that HgCl_2_ solution should not be used in udder disinfection; it would be appropriate to use boric acid solution (5% boric acid in hot water) instead, and cheeses should not be covered with hemlock (BOA, 827‐70).

*June 16, 1905 Dated Archival Document*: This document states that in one of the villages of Skopje in Macedonia, a merchant sold spoiled butter and spoiled sourdough cheese in the market. The spoiled cheeses were confiscated and taken to Skopje Municipality, where the seller was fined for selling spoiled cheese under inappropriate conditions. Other market vendors also reported that the person in question was selling spoiled cheese (BOA, 109‐58).

*September 14, 1905 Dated Archival Document*: This document states that there were complaints about negligence in the production of cheeses and other foods in Bulgaria and that measures should be taken by making controls on this issue (BOA, 9‐864).

### 3.2 Selling price of cheese in the Prime Ministry Ottoman Archives

*May 2, 1803 Dated Archival Document*: This document states that a cheese merchant in the Eminönü neighborhood of İstanbul was selling fresh cheese for 40–45 money instead of 36 money per okka, and that measures should be taken because he was causing losses to the public (BOA, 80‐3965).

*January 25, 1812 Dated Archival Document*: This document states that although the price of fresh meadow cheese in İstanbul was 23 money per okka, a greengrocer in the Eminönü neighborhood was selling it for 40 money, causing losses to the public, and measures should be taken (BOA, 76‐3792).

*September 17, 1830 Dated Archival Document*: This document states that in settlements such as Bozcaada in Çanakkale province, sellers who sold cheese above the price set by the municipality were fined (BOA, 31‐1533).

*June 9, 1857 Dated Archival Document*: This document states that a municipal circular was issued in İstanbul setting the prices of kashkaval cheese (BOA, 310‐21).

*May 3, 1871 Dated Archival Document*: In this document, it was stated that fresh cheese to be sold by Skopje artisans in Macedonia would be sold for 70 money per okka (BOA, 67‐34).

*July 20, 1872 Dated Archival Document*: This document states that in Skopje Municipality in Macedonia, due to the decrease in the milk obtained from sheep in dairies, dairy shopkeepers requested the municipality to re‐determine the price of milk and cheese (BOA, 68‐170).

*October 19, 1878 Dated Archival Document*: This document states that a 10 fine was imposed on a grocer who sold cheese at an exorbitant price in a village in Romania (BOA, 72‐166).

*March 23, 1880 Dated Archival Document*: This document states that the municipality of Skopje in Macedonia determined the sale prices of various meat, oil, grain, pulses, cheese, hay, wood, halva, etc. (BOA, 74‐113).

## 4 DISCUSSION

In the Ottoman Empire, after 1826, municipal police officers and health officers were responsible for food control, and after 1844, food analysis was carried out in chemistry laboratories. Suspicious cheese samples were taken by municipal police officers and sent to the laboratory for analysis. In the Ottoman Empire, customs were responsible for the control of food during its importation or circulation within the country. Domestic customs were responsible for the movement of food within the country (sanitary control and taxation). External customs were responsible for the control (sanitary control and taxation) of goods at border crossings in interstate trade. Internal customs began to be abolished in Europe in the late 18th century and were completely abolished in the mid‐19th century. In the Ottoman Empire, internal customs continued until the early 20th century. In 1801, the number of internal Ottoman customs was over 100 (Kütükoğlu, 1996).

In the 19th century, there were important scientific developments in the microbiological analysis of foods, and new methods started to be applied. The Ottoman Empire followed the developments in science in the identification of foodborne diseases and sent students to universities in other countries for education on this subject. In an archival document dated October 17, 1894 (BOA, 2‐46), it is stated that the Ottoman Empire sent students to Germany to receive training in agriculture and that four people who had previously attended a university in Berlin received training in agricultural subjects such as oil and cheese production, horticulture, and animal husbandry, and that the student's education was extended.

At the time of the research, the phenomenon we call food poisoning today was referred to with different words. In this period, the terms spoiled cheese and sickness from cheeses were used for the cheeses that caused the disease. This way of expression is correct because the discovery of microorganisms was made in the late 17th century and the expression of microbial food poisoning cannot be fully expected in this period. In the Ottoman Empire, there was no distinction between inedible spoilage of cheeses and causing illness; both conditions were referred to as spoiled cheese. In addition to referring to cheese spoilage in these years, the documents also indicate that measures were taken against spoilage (or disease) agents at cheese sales points and production areas. A document dated May 28, 1901 (BOA, 2490‐50) reported that cheese production conditions should be controlled and cheese containers should be tinned. During the research period, it was determined that cheese or other foods were kept in copper containers (Table 1). During these periods, illnesses caused by copper containers were realized (Kocacık & Mat, 2014). Again, in the document dated February 10, 1905 (BOA, 958‐32), it was stated that the udder of the animals should be cleaned and disinfected before milking, as disease agents could be transmitted from the udder of the animals. In a period when antimicrobials were not used effectively, these measures reduced the emergence and spread of cheese‐borne diseases.

**TABLE 1:** BAO documents specifying cheese production conditions.

	BOA number	Document number
Production condition	2490‐50, 704‐63, 242‐112, 958‐32, 827‐70, 9‐864	6
Supervision of sales conditions	68‐63, 2490‐50, 242‐112, 109‐58	4
Used copper container	2490‐50	1
Used hemlock	958‐32, 827‐70	2
Using rennet	813‐35	1
Analysis	80‐36, 668‐54, 2503‐22, 674‐99, 225‐10, 704‐63, 2503‐22, 301‐67, 301‐51, 294‐19, 813‐35, 913‐42	12
Chemical and bacteriological analysis	301‐67, 294‐19	2

Since ancient times, cheese has been the main food source in every society. For these reasons, cheese poisoning is the most prominent foodborne illness (Taşçıoğlu, 2022). In the Ottoman archive, 31 documents suitable for the content of the study were identified. Of these, spoilage was mentioned in 12 documents (Figure 1), disease in 9 documents (Figure 1), the use of rennet in one document (Table 1), and punishment for spoiled cheese in 7 documents. The first document mentioning spoiled cheese in the period under study is dated June 8, 1872 (BOA, 68‐63), and illness from spoiled cheese is mentioned in a document dated April 21, 1885 (BOA, 80‐36). Only two documents mention vomiting as a symptom of illness caused by cheese during the period under investigation (Figure 1). The establishment of laboratories for food analysis began in Europe and the Ottoman Empire in the mid‐19th century. The existence of microorganisms was determined after the discovery of the microscope (1670), and the expression of microorganisms as foodborne disease agents was realized in the following years. The first mention of bacteria originating from cheese in Ottoman state documents was found in a document dated May 9, 1903 (BOA, 704‐63). There were no significant (*p* > .05) differences between the number of documents indicating diseases and spoilage. The number of archive documents indicating diseases significantly (*p* < .05) differed from the number of archive documents indicating disease agents, death, vomiting, swelling, and death. The number of archive documents indicating spoilages significantly (*p* < .05) differed from the number of archive documents indicating disease agents, death, vomiting, swelling, and death.

From the Ottoman archive documents, the names “feta cheese, kashar cheese, tulum cheese, kashkaval cheese, Albanian cheese, tongue cheese, tin cheese, meadow cheese, brined cheese, plain cheese, highland cheese, Italian cheese, curd cheese, salted cheese, Balkan cheese, sourdough cheese, Şarköy kashar cheese” were identified (Table 2). It was determined that the disease was mostly caused by fresh meadow cheese (41.7%) (Table 2). Sourdough cheese (8.3%) and pickled cheese (8.3%) were the other diseases associated with cheeses. Currently, official reports on food poisoning are not kept in Turkey. However, there are many studies on the level of contamination and presence of pathogenic microorganisms in many foods, such as cheese (Bingöl, 2016; Çağlar et al., 1996; Gürler, 2009; Kalkan & Halkman, 2006; Kaynar, 2011; Tirsi, 2016). It has been reported that approximately 1/3 of food poisonings in the world are caused by foods contaminated with enterotoxigenic *Staphylococcus aureus* (Küçükçetin & Milci, 2008). *S. aureus* was detected between 530 colony‐forming units (cfu)/g in feta cheese in Van (Coşkun & Öztürk, 2000), between 60 and 1.3 × 10^3^ cfu/g in feta cheese in Erzurum, 550 cfu/g in fresh feta cheese in Elazığ, between 2.2 × 10^4^ and 4.1 × 10^7^ cfu/g in fresh feta cheese in Antalya, and between 20 and 1.0 × 10^6^ cfu/g in herbed cheese in Van (Küçükçetin & Milci, 2008). These results indicate that many cheeses offered for sale in Turkey have a risk of carrying *S. aureus* above 10^5^ cfu/g. The presence of *S. aureus* at this level in cheeses indicates that there is a high probability of enterotoxin production and cheese poisoning.

**TABLE 2:** Cheese varieties and status of cheeses included in BAO documents.

Cheese	BOA number	Cheese situation	Number of archived documents
Fresh meadow cheese	2490‐50, 704‐63, 242‐112, 958‐32, 827‐70	Causing disease	5
Fresh meadow cheese, feta cheese, pickled cheese, and sourdough cheese	704‐63, 674‐99, 225‐10, 109‐58	Spoiled cheese and controlling it	4
Curd cheese, kashar cheese, Şarköy kashar cheese, and salted cheese	2503‐22	Unhealthy cheeses	1

In the study, there were 12 documents indicating that samples were taken from the cheeses suspected to be contaminated and sent to the laboratory for analysis, and two of these documents indicated that chemical and bacteriological analyses should be carried out. It was determined that the sale of spoiled cheeses was stopped and necessary inspections were carried out at the production sites. Those who sold and produced spoiled cheese were fined by the municipalities. In the research, it was determined that fines were imposed on 3 documents due to inappropriate production and selling conditions of cheeses. Cheese spoiled by the swelling of the tin container was mentioned in two documents (Figure 1). In addition to the importance of producing cheeses under appropriate conditions, it was also determined that measures were taken to prevent cheese whey from causing environmental pollution. In the document dated August 20, 1883 (BOA, 78‐69), it is stated that Skopje Municipality fined a grocer 5 times for polluting the environment by pouring cheese whey on the street. There were no significant (*p* > .05) differences among archive documents indicating disease agents, death, vomiting, swelling, and death.

It is understood from the archival documents that the authorities in the Ottoman Empire had a sufficient level of awareness about the fact that foods carried disease agents, and as a result, people became ill. In the document dated May 8, 1909, it was stated that the sale of dairy products brought from Malta and Algeria was banned for a while due to the tainted milk, cheese, and meat milked from goats raised in Malta and sheep raised in Algeria (BOA, 3544‐265798). Again, in the document dated October 10, 1886, it was stated that the cheese and animals on the ships in Kavak state of İstanbul were quarantined for a while due to the risk of disease transmission, and the passengers on the ships were quarantined for disease controls (BOA, 13‐17). In addition, during the period under investigation, it was emphasized that poisonous herbs would cause illness when they came into contact with cheese, and it was understood that such plants should not be used in cheese production and were even banned. In two documents dated May 7, 1905 and February 10, 1904, it was stated that when fresh meadow cheeses were covered with hemlock, poisonous substances could pass into the cheese and that cheeses should not be covered with these herbs (Table 1).

During the period under investigation, the word bacteria was used as a disease agent in only two documents (Figure 1). In the 20th century, it was still unclear which microorganism was the cause of food poisoning in the world. In the Ottoman Empire, the term bacteria started to be used only for food diseases at the beginning of the 20th century. During the period of the study, only vomiting was stated as a symptom of two cheese diseases in two documents (Figure 1).

## 5 CONCLUSION

It was determined that legal measures had been taken in the Ottoman Empire to prevent cheese counterfeiting and to produce and sell safe cheeses in line with the microbiological and chemical knowledge of the period. Considering that cheese spoilage and cheese‐induced poisoning are still seen at an insignificant level in many countries today, it has been determined that both the Ottoman Empire and the Republic of Turkey, which was established in 1923, made legal arrangements for the prevention of food spoilage and foodborne diseases with effective measures in the 19th and 20th centuries. The results of our research have shown that it is necessary to reveal the situation of the past time by conducting research on food in the past to shed light on past approaches to the present. The identification and compilation of early food poisonings and practices will provide a unique opportunity to apply a range of scientific methods that will help further elucidate regional/social habits and patterns and resolve imbalances and limitations in historical evidence.

## AUTHOR CONTRIBUTIONS

**Ayşe Erkmen:** Conceptualization (equal); formal analysis (equal); investigation (equal); methodology (equal); visualization (equal); writing – original draft (equal); writing – review and editing (equal). **Nevim Tüzün:** Formal analysis (equal); investigation (equal); methodology (equal); resources (equal). **Osman Erkmen:** Investigation (equal); methodology (equal); project administration (equal); software (equal); supervision (equal); validation (equal); writing – original draft (equal); writing – review and editing (equal).

## CONFLICT OF INTEREST STATEMENT

All authors declare that they have no conflicts of interest.

## PRACTICAL APPLICATION

The increase in cheese production has led to the spread of cheese‐borne diseases. It is necessary to reveal the situation of the past time by conducting research on food in the past to shed light on past approaches to the present. Measures to prevent food spoilage or disease in the past have continued to be developed until today and have enabled modern measures to be taken today.

## Data Availability

The data presented in this study are available on request from the corresponding author.
